# Pain, Discomfort, and Functional Impairments When Retracting Upper Anterior Teeth Using Two-Step Retraction With Transpalatal Arches Versus En-Masse Retraction With Mini-implants: A Randomized Controlled Trial

**DOI:** 10.7759/cureus.33524

**Published:** 2023-01-09

**Authors:** Mudar M Mousa, Salma Al-Sibaie, Mohammad Y Hajeer

**Affiliations:** 1 Orthodontics, University of Damascus Faculty of Dentistry, Damascus, SYR; 2 Orthodontics, Dr. Aburas Dental Center, Dubai, ARE

**Keywords:** speech, functional impairment, questionnaire, discomfort, pain, two-step retraction, en-masse retraction, anchorage, transpalatal arches, mini-implants

## Abstract

Background

This study aimed to evaluate the levels of pain and discomfort associated with employing mini-implants as a temporary skeletal anchorage device compared to the traditional transpalatal arches (TPAs) during upper anterior teeth retraction in patients with upper dentoalveolar protrusion and to determine the level of acceptance of both techniques among patients.

Methodology

The study sample consisted of 38 patients (29 women and nine men) with an average age of 21.7 years. The patients were randomly and equally distributed into two groups. In the first group: upper anterior teeth were en-masse retracted using mini-implants (the TAD group), whereas, in the second group, TPAs were used during the two-step retraction of upper anterior teeth (the TPA group). Standardized questionnaires were distributed to all patients after 24 hours of mini-implant application. The questionnaire asked the patients to rate their pain perception, swelling sensation, eating difficulties, talking impairments, and cleansing difficulties on a four-point Likert scale on the third-day, one-week, two-week, and one-month follow-ups after the anchorage application. Wilcoxon matched-pairs signed-rank tests were used to evaluate intragroup changes, whereas Mann-Whitney U tests were employed to examine intergroup differences.

Results

Patients in the TAD group had higher pain and swelling levels than those in the TPA group, and differences were statistically significant at the first three assessment time points. The differences between the two groups were statistically insignificant regarding eating and talking difficulties, whereas differences were statistically significant for brushing difficulties. These impairments decreased to almost normal levels after one month of treatment initiation.

Conclusions

TPAs, when used for anchorage in the two-step retraction technique, were less problematic compared to mini-implants with en-masse retraction, where the sensation of pain or swelling around the mini-implants did not last for more than a week. The difficulties of cleaning, chewing, and speaking in the presence of mini-implants were temporary and mostly disappeared within two weeks of mini-implant application.

## Introduction

Pain is defined as an unsatisfactory sensation that is caused by internal or external sources or associated with actual damage to various tissues [1]. Fear of pain is the primary reason for orthodontic treatment discontinuation [2]. To understand the extent to which patients cooperate and accept a treatment method, it is insufficient to only study the effectiveness and feasibility of the method; it is necessary to determine the level of pain patients can tolerate when using this method [3].

The orthodontic treatment causes varying degrees of pain and discomfort to the patient, leading to decreased cooperation and may result in treatment discontinuation [4]. To date, a large number of studies have been conducted on patient-reported outcome measures (PROMs) associated with various orthodontic procedures, such as pain during orthodontic separation [5], discomfort and functional impairments when using removable and fixed expansion appliances [6], acceptance of the functional and removable appliances [7,8], oral health-related quality of life (OHRQoL) of patients undergoing clear aligner treatment versus fixed appliances [9], speech problems and oral functions when using lingual brackets versus labial brackets [10,11], and the acceptability of different retainers at the end of orthodontic treatment [12].

Anchorage is one of the most important biomechanical principles in orthodontics. It ensures that the supporting teeth do not move as other teeth are being moved [13]. Several methods have been proposed to assure good anchorages, such as headgear [14], transpalatal arches (TPAs), with or without a Nance button [15], lingual arches, bonding of second molars, or intermaxillary elastics [16], and most recently, mini-implants [17].

Extraction of the upper premolars is one of the most common procedures to camouflage class II division 1 malocclusion. Closure of the extraction space can be achieved by a one- or two-step retraction of the upper anterior teeth [17]. Two-step retraction involves dividing the retraction of the anterior teeth into two separate steps; this technique puts less load on the anchorage unit. This, therefore, requires medium anchorage, which can be achieved in various ways, including the use of TPAs [18]. In contrast, in the en-masse retraction, canines and incisors are retracted as one block, thereby shortening the treatment time. However, it requires absolute anchorage; this can be done using mini-implants, which have become more popular recently [19].

The use of mini-implants is generally associated with pain and discomfort from the first day of insertion. Lee et al. [20] reported that patients experienced mild to moderate levels of pain on the first day following the insertion, with the pain decreasing on the third day and reaching a mild level on the seventh day. Pithon et al. [21] noted a mild level of pain for the use of mini-implants after 28 days of insertion.

In addition, the use of mini-implants is associated with functional impairment; Lee et al. [20] reported that 36% of their patient population experienced moderate chewing difficulties, while, in Kuroda et al. [22], the patients experienced mild chewing difficulty [22]. However, in Feldmann et al. [3], the only randomized controlled clinical trial (RCT) to evaluate the PROMs associated with using TPA for anchorage enhancement [3], found that their patients experienced a moderate level of pain two days after the installation of a TPA, which decreased to a mild level after seven days and then decreased completely. However, research on the functional impairments during the long-term use of TPAs seems to be scarce in the current literature.

Numerous published studies have examined the extent of pain and discomfort associated with the insertion and removal of mini-implants during orthodontic treatment. However, few studies have examined the short- or long-term patient-reported outcomes during the clinical use of mini-implants (i.e., from three months up to 12 months after anterior teeth retraction). Therefore, our RCT aimed to compare the levels of pain, discomfort, functional impairment, and acceptability of patients with class II division 1 malocclusion when mini-implants are used for maximum (or absolute) anchorage during en-masse retraction versus when TPAs are used during the two-stage retraction.

## Materials and methods

Study design and settings

This RCT was conducted on patients with a maxillary protrusion at the Department of Orthodontics at the University of Al-Hama (currently the University of Hama), Hamah, Syria. This study was registered at Clinical Tial.gov with the following ID (NCT05652244). It was approved by the Local Research Ethics Committee of the University of Hama (Approval no. UHDS-29182015PG/SRC1108) and was funded by the University of Hama Postgraduate Research Budget (Reference number: UHDS-6013_2015DENRB).

Sample size calculation

Minitab® Version 15 (Minitab Inc., State College, Pennsylvania, USA) was used to estimate the sample size. A 20-mm difference in pain perception on a visual analog scale (VAS) was chosen as the minimum clinically significant difference requiring detection between the two groups (with a standard deviation of pain perception after seven days of insertion of 16.17 mm, assessed on a 100-mm VAS according to a previous study) [20]. Using an independent-samples t-test with a power of 95% and a significance level of 5%, 19 patients were required in each group.

Patient recruitment and eligibility criteria

Records from the department of orthodontics were reviewed. After clinical and radiographic examination, 45 patients who met the inclusion criteria were informed about the details of the study and received a detailed explanation of the two treatment methods; an information sheet was presented to them, and their informed consent was obtained. Of the total number of patients, 42 agreed to participate in this study. According to the sample size calculation, 38 (29 females and nine males) patients were randomly selected. Then, the patients were randomly and equally distributed into two groups.

**Figure 1:**
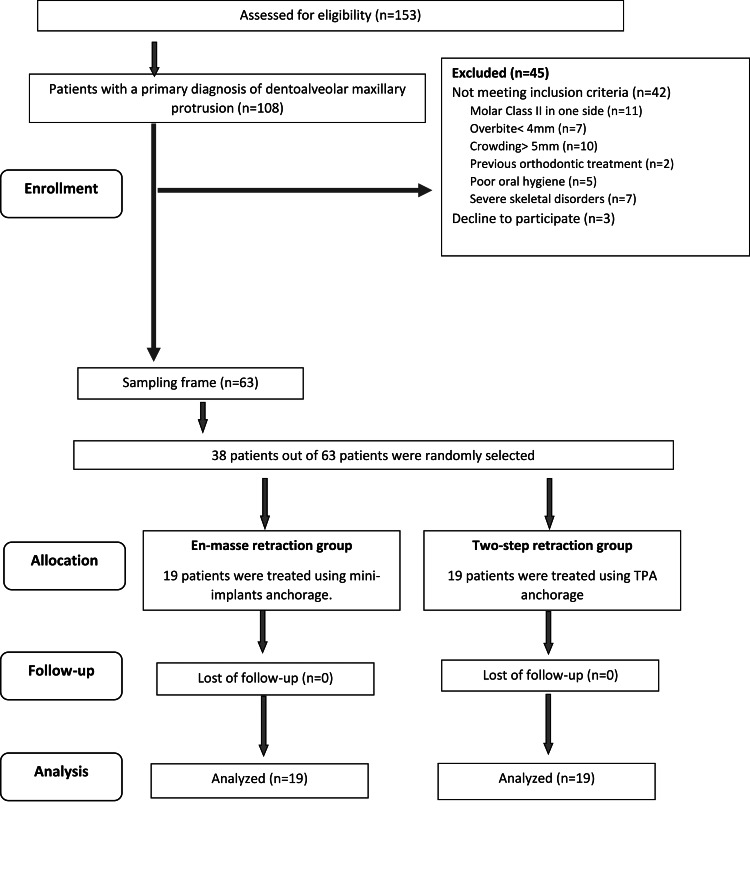
The Consolidated Standards of Reporting Trials (CONSORT) flow diagram of patients' enrolment, allocation, follow-up, and entry into data analysis.

The inclusion criteria included the following: (1) patients with class II division 1 malocclusion; (2) overjet of more than 5 mm; (3) a normal overbite (more than 0 mm and less than 4 mm); (4) skeletal class II relationship (4 < ANB < 10); (5) normal or increased anterior facial height; (6) well-aligned maxillary teeth with minimal crowding (≤4 mm according to Little’s index); and (7) complete permanent dentition (except for the third molars). Exclusion criteria were as follows: (1) previous orthodontic treatment; (2) poor oral hygiene; (3) any craniofacial syndromes; and (4) medical conditions that affect tooth movement. The Consolidated Standards of Reporting Trials (CONSORT) flow diagram of patient recruitment, follow-up, and inclusion for data analysis is shown in Figure 1.

Randomization and allocation concealment

Minitab® Version 15 (Minitab Inc., State College, Pennsylvania, USA) was used to generate the random number sequence for patient assignment into two groups. The allocation sequence was concealed using a series of random numbers; then, the allocation sequence was masked using numbered, opaque, and sealed envelopes, which were opened only after the extraction of premolars. An academic person not involved in this study was asked to perform the sequence generation and participants’ enrollment and assignment to an intervention. The 38 patients included after considering the inclusion and exclusion criteria were divided randomly into two groups. Blinding was limited to the data analysis only, and it did not apply to either participants or practitioners.

Specifications of the devices used

The orthodontic treatment was performed by the principal investigator (S.A.S) under the supervision of one of the coauthors (M.Y.H) at the orthodontic department of Hama University Faculty of Dentistry.

MBT prescription brackets with a 0.022-inch slot height (Mini-Taurus, RMO®, Denver, Colorado, USA) and bands (with welded TPA for the two-step retraction group) were used for the upper first molars (TruForm, RMO®, Denver, Colorado, USA). Leveling and alignment were conducted with the following archwires sequence: 0.0155- inch twisted wire (Supra-flex, RMO®, Denver, Colorado, USA), 0.0175-inch twisted wire (Tri-flex, RMO®, Denver, Colorado, USA), nickel-titanium wires 0.016 inch × 0.022 inch, 0.017 inch × 0.025 inch (Orthonol, RMO®, Denver, Colorado, USA), and finally, 0.019 inch × 0.025 inch stainless steel wires (True-chrome, RMO®, Denver, Colorado, USA) with brass hooks soldered distal to the lateral incisors for mini-implant patients or without hooks for TPA anchorage patients.

The first premolars were extracted for all patients after leveling and alignment in the department of oral and maxillofacial surgery, Hama University Faculty of Dentistry. Subsequently, for the en-masse retraction group, mini-implants were applied after one week of extraction.

First group: two-step retraction of upper anterior teeth with TPAs

**Figure 2:**
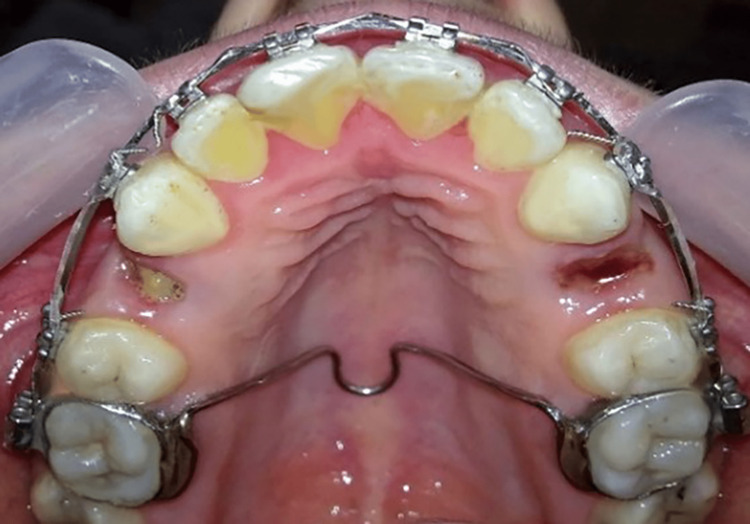
Passive transpalatal arches (TPAs) soldered to the upper molar bands in the first group. TPAs were used as a source of anchorage.

Neutral TPAs were placed at the level of the molars, welded to the bands of the maxillary first molars, and designed by the same laboratory with a 0.9-mm-diameter stainless steel wire at a distance of 2 mm from the palate to prevent implantation in the soft tissues, and with Coffin loop in the center (Figure 2).

The canines were moved distally using closed elastic chains. After achieving the class I canine relationship, they were grouped with the posterior units, and the four anterior incisors were then retracted [23]. Patients were examined every three weeks until the completion of retraction with a good incisor relationship or until the spaces lateral to incisors were closed (end of observation).

Second group: en-masse retraction of upper anterior teeth with mini-implants

**Figure 3:**
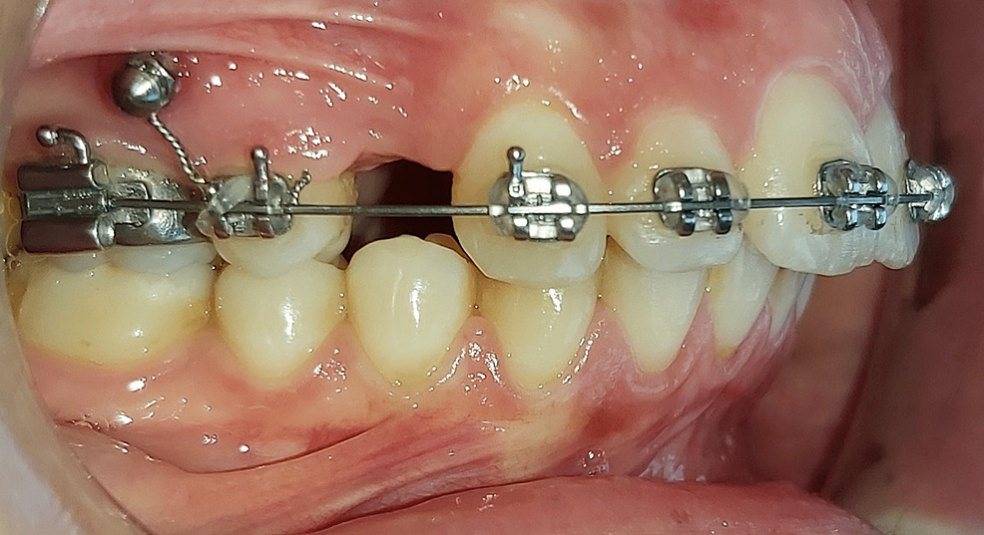
The fixed appliance on the upper dental arch with a mini-implant inserted between the second premolar and the first molar as a source of anchorage.

Self-drilling titanium orthodontic mini-implants (1.6 mm in diameter, 6 mm in length; OSAS, Dewimed®, Tuttlingen, Germany) of the head-mounted model with a hole for inserting the ligature wires when needed (Figure 3).

After the administration of local anesthesia, the mini-implants were inserted bilaterally between the roots of the maxillary second premolars and first molars at the mucogingival junction, approximately 8-10 mm above the archwires. To ensure no mesial movement of the posterior teeth, the maxillary second premolar and first molar were rigidly attached to the miniscrew with a ligature wire. The en-masse retraction was obtained with two elastomeric chains (Energy Chain Closed; RMO®, Denver, Colorado, USA) placed between the mini-implants and the soldered hooks in a direction approximately parallel to the occlusal plane; a force of 250 g was applied on each side to perform the en-masse retraction. The retraction was stopped when a class I canine relation was achieved, a good incisor relation was obtained, or spaces lateral to canines were closed.

Outcome measures: questionnaires

A standardized questionnaire was used to assess pain and discomfort levels during the treatment. This questionnaire was based on the one that Sergl et al. [24] had used and Saleh et al. [8] and Khattab et al. [10] had further modified. We also made a few changes to the adopted questionnaire.

Questionnaires were administered at five assessment points after mini-implant insertion: at 24 hours (T1), three days (T2), one week (T3), two weeks (T4), and one month (T5). The questionnaire contained five questions about patients' perception of (1) pain, (2) swelling, (3) difficulty in chewing, (4) speech problems, and (5) difficulty in appliance and oral cleaning (Appendix 1). The answers were provided using a four-point Likert scale: 1: not at all; 2: little; 3: much; 4: very much. Then, the questionnaires were given to the patients, who answered them in the treatment chair after they were made aware of the options available for each question. The patients chose the option they deemed the most appropriate.

Before using the questionnaire in the main study, a pilot study was conducted to identify any comprehension difficulties and additional complaints that might result from using mini-implants or TPAs during the active treatment phase. The pilot study sample consisted of four participants with class II division 1 malocclusion; of these four participants, two were treated with fixed appliances with mini-implant anchorage and two were treated with fixed appliances using TPA anchorage. These questionnaires were completed seven, 14, and 30 days following the mini-implant placement. According to the pilot study, no modification to the questionnaire was required.

## Results

Changes in the PROMs over time in each group

Pain Experience

After 24 mini-implant insertions, 79% of all patients in the mini-implants group reported that they felt moderate to severe pain; this percentage decreased significantly at three days (T2; P = 0.002). After one month of insertion (T5), 84.21% of the patients felt mild or no pain (Table 1). On the other hand, 94.74% of patients in the TPA group felt mild or no pain 24 hours after the insertion; the level of pain increased insignificantly at the next three assessment times (Table 2).

**Table 1:** Descriptive statistics of patient-centered variables for the questions at five assessment time points in the en-masse retraction group using a four-point Likert scale.

Question	1 (Not at all)	2 (Little)	3 (Much)	4 (Very much)
Q1: Pain at the site of the mini-implants				
T1	0	21.05	31.58	47.37
T2	15.79	31.58	47.37	5.26
T3	5.26	42.11	31.58	21.05
T4	47.37	26.32	26.32	0
T5	57.89	26.32	15.79	0
Q2: Swelling around the mini-implants				
T1	10.53	73.68	10.53	5.26
T2	42.11	36.84	21.05	0
T3	36.84	36.84	26.32	0
T4	84.21	10.53	5.26	0
T5	73.68	21.05	5.26	0
Q3: Chewing difficulty due to the mini-implants				
T1	10.53	21.05	57.89	10.53
T2	31.58	42.11	21.05	5.26
T3	26.32	21.05	36.84	15.79
T4	31.58	47.37	21.05	0
T5	63.16	36.84	0	0
Q4: Speech discomfort due to the mini-implants				
T1	26.05	26.32	47.37	5.26
T2	31.58	47.37	21.05	0
T3	52.63	36.84	10.53	0
T4	68.42	26.32	5.26	0
T5	78.95	21.05	0	0
Q5: Difficulty in cleaning around the mini-implants				
T1	10.53	21.05	42.11	26.32
T2	15.79	36.84	31.58	15.79
T3	5.26	36.84	36.84	21.05
T4	26.32	52.63	21.05	0
T5	47.37	42.11	5.26	5.26

**Table 2:** Descriptive statistics of patient-centered variables of the questions at five assessment time points in the two-step retraction group using a four-point Likert scale.

Question	1 (Not at all)	2 (Little)	3 (Much)	4 (Very much)
Q1: Pain after the TPA application				
T1	63.16	31.58	5.26	0
T2	73.68	26.32	0	0
T3	73.68	26.32	0	0
T4	78.95	21.05	0	0
T5	100	0	0	0
Q2: Swelling around the TPA				
T1	68.42	31.58	0	0
T2	73.68	26.32	0	0
T3	89.47	10.53	0	0
T4	100	0	0	0
T5	100	0	0	0
Q3: Chewing difficulty due to the TPA				
T1	52.63	36.84	10.53	0
T2	52.63	36.84	10.53	0
T3	21.05	36.84	42.11	0
T4	36.84	42.11	21.05	0
T5	52.63	42.11	5.26	0
Q4: Speech discomfort due to the TPA				
T1	26.32	26.32	47.37	0
T2	31.58	63.16	5.26	0
T3	74.36	52.63	0	0
T4	52.63	47.37	0	0
T5	68.42	31.58	0	0
Q5: Difficulty in cleaning around the TPA				
T1	31.58	26.32	36.84	5.26
T2	36.84	42.11	15.79	5.26
T3	36.84	57.89	5.26	0
T4	57.89	42.11	0	0
T5	73.68	26.32	0	0

Swelling

After 24 hours of the mini-implant insertion, 73.68% of the patients reported mild swelling at the site of the mini-implant. There was a significant decrease at two weeks and one month compared with the first assessment time (T4, P = 0.002; T5, P = 0.004). None of the patients experienced severe or moderate swelling in the areas surrounding the TPA at each assessment time. The level of perceived swelling in the neighboring tissues was close to zero in all patients after two weeks of insertion.

Chewing Difficulty

At 24 hours of the mini-implant insertion, 68.42% of the patients in the mini-implants group experienced moderate to severe discomfort while eating; this percentage decreased significantly at three days (T2; P = 0.006). After one month of mini-implant insertion, all patients experienced no or mild discomfort. In the TPA group, after 24 hours and three days, 89.47 % of the patients reported no or mild chewing discomfort. However, 94.74% of the patients felt mild or no difficulties after one month, with no significant difference from the difficulties experienced at T1.

Speech Discomfort

At 24 hours of the mini-implant insertion, 47.37% of the patients in this intervention group experienced moderate discomfort in speaking; this percentage significantly decreased to 21.05% at three days and 10.53% at seven days (T2, P = 0.004; T3, P = 0.025). Moreover, after one month, all patients felt mild or no discomfort while speaking. On the other hand, for the TPA group, 47.37% of the patients felt moderate discomfort at 24 hours, and this percentage decreased significantly at three days and one week (5.26%, 0%, respectively; P = 0.009; Table 2).

Cleaning Difficulty

After 24 hours of the mini-implant insertion, 68.43% of the patients felt moderate to severe difficulty in cleaning around the mini-implants, and this percentage decreased significantly at two weeks and one month (P = 0.017 and P = 0.005, respectively). About 36.84% of the patients in the transpalatal arch group reported experiencing mild difficulty in cleaning after 24 hours of the procedure. At seven days, 94.74% of the patients had no difficulty cleaning.

Differences between the two groups in patients' responses to the questionnaire

The perception of pain in the mini-implant group was moderate to severe after 24 hours, which then reduced to mild or moderate at three days, seven days, and two weeks (T2, T3, and T4; mean = 1.42, 1.26, 1.26, and 1.21, respectively; Table 3). However, pain perception in the TPA group was mild to moderate after 24 hours, which then reduced to mild at three days, seven days, and two weeks (T1, T2, T3, and T4; mean = 1.42, 1.26, 1.26, and 1.21, respectively). Pain sensation was significantly greater in the mini-implant group (T1, T2, T3; P < 0.001, and T4; P = 0.023).

**Table 3:** The mean values of patient responses in the two groups at the five assessment time points and the p-values of significance of mean differences between the two groups. SD: standard deviation; NS: non-significant; NA: not applicable; *P < 0.05, **P < 0.01, ***P < .001

Assessment time	Question	En-masse retraction group (G1)		Two-step retraction group (G2)		Significance of mean difference (G2-G1)	
		Mean	SD	Mean	SD	P-value	Significance
First	1	3.26	0.81	1.42	0.61	<0.001	***
	2	2.11	0.66	1.32	0.48	<0.001	***
	3	2.68	0.82	1.58	0.68	<0.001	***
	4	2.37	0.90	2.21	0.86	0.615	NS
	5	2.84	0.96	2.16	0.96	0.039	*
Second	1	2.42	0.84	1.26	0.45	<0.001	***
	2	1.79	0.79	1.26	0.45	0.027	*
	3	2.00	0.88	1.58	0.90	0.055	NS
	4	1.90	0.74	1.74	0.56	0.535	NS
	5	2.47	0.96	1.90	0.88	0.061	NS
Third	1	2.68	0.89	1.26	0.45	<0.001	***
	2	1.90	0.81	1.11	0.32	0.001	**
	3	2.42	1.07	2.21	0.79	0.518	NS
	4	1.58	0.69	1.53	0.51	1.000	NS
	5	2.74	0.87	1.68	0.58	<0.001	***
Fourth	1	1.79	0.86	1.21	0.42	0.023	*
	2	1.21	0.54	1.00	0.00	NA	
	3	1.90	0.74	1.84	0.77	0.826	NS
	4	1.37	0.60	1.47	0.51	0.422	NS
	5	1.95	0.71	1.42	0.51	0.019	*
Fifth	1	1.58	0.77	1.00	0.00	NA	
	2	1.32	0.58	1.00	0.00	NA	
	3	1.37	0.50	1.53	0.61	0.457	NS
	4	1.21	0.42	1.32	0.48	0.479	NS
	5	1.68	0.82	1.26	0.45	0.077	NS

Most patients in the mini-implant group reported mild to moderate perceptions of swelling in the tissues around the mini-implants at 24 hours, three days, and seven days (T1, T2, and T3; mean = 2.11, 1.79, and 1.90, respectively). Meanwhile, these perceptions in the TPA group at all assessment time points were very mild to mild (T1, T2, T3, T4, and T5; mean = 1.32, 1.26, 1.11, 1.00, and 1.00, respectively). The differences between the two groups were significant and greater in the mini-implant group at 24 hours, seven days, and two weeks (T1, T2, and T3; P < 0.001, P < 0.01, and P < 0.05, respectively). Difficulty chewing was significantly greater in the en-masse retraction group after 24 hours, with a mean value of 2.68, compared to the two-step retraction group, with a mean value of 1.58. However, the two groups had no significant differences at all the remaining time points. Most patients in both groups had mild to moderate discomfort while speaking after 24 hours of the application; the discomfort decreased to very mild at the remaining time points, with no significant differences between the two groups. The difficulty of cleaning around the mini-implants was significantly greater at 24 hours, seven days, and two weeks as compared to the areas around the TPAs, but the differences were insignificant after three days and one month.

## Discussion

The degree of acceptance of orthodontic treatment is measured by the amount of discomfort the patient experiences during or after the treatment [24]; after the application, the patient may experience a significant amount of discomfort, such as pressure, tension, and pain [25]. The increase in the discomfort caused by the orthodontic appliance negatively affects the acceptance of the treatment and the degree of patient cooperation [26]. Although there is a large number of studies on the dental-alveolar and skeletal effects of retraction [17,23], no study has studied patient-reported measures after retraction using different anchoring methods.

Pain perception

The results of this study showed that pain levels were high after 24 hours of mini-implant insertion. This is because 78.95% of the patients in this intervention group reported experiencing severe or moderate pain after 24 hours of the mini-implant placement due to the puncture in the underlying gingival and bone tissues caused by the self-drilling mini-implants, which resulted in the crushing of part of the surrounding alveolar bone and periodontal soft tissues. However, over time, and specifically over the next three days, only 5.26% of the patients reported severe pain, as the tissues surrounding the mini-implants had begun to recover; subsequently, 21.05% of the patients reported severe pain after one week, which was the time of applying the retraction forces (i.e., the power chain). Then, about 15% of patients reported that they continued to experience moderate pain after one month due to the reactivation of the retraction forces.

During the first two weeks, the pain level was greater in the en-masse retraction group, which may be because of the insertion of the mini implants, and the retraction force was greater in the en-masse retraction group than the two-step retraction group (250g and 150g, respectively). However, the differences between the two groups gradually decreased and were no longer significant after one month of the applications.

The results of this study differ from those of Kuroda et al. [22], who compared two types of mini-implants (the first type was 7-11 mm in length and 2-2.3 mm in diameter, and the second type was 6-12 mm in length and 1.3 mm in diameter). The researchers reported that 50% of the patients did not experience pain at any point during the treatment period. This difference in pain perceptions is the result of the use of a smaller-diameter mini-implant by Kuroda et al. [22] as compared to that used in this present study. Their study used two-step retraction with retraction forces ranging from 50g to 200g.

Swelling

The study results showed that a high percentage of patients (73.68%) felt mild swelling in the soft tissues surrounding the mini-implants after 24 hours of application; subsequently, the degree of swelling decreased over the next six days but increased again at the end of the first week due to the irritation caused by the extension of the elastic chains in continuous contact with the gingival tissues located under these chains. Over the weeks following the activation, the tissues began to recover, and the swelling gradually decreased and disappeared within approximately two weeks of the application.

Swelling levels were significantly higher in the en-masse retraction group than in the two-step retraction. This was because, in the two-step retraction group, the extension of the elastic chains was in direct contact with a small gingiva area, whereas, in the en-mass retraction group, the extension of the elastic chains was in greater contact with the gingiva and was near the mucogingival junction, which may be more sensitive to irritation.

The results of this study differ from those of Kuroda et al. [22], who found that most patients reported no swelling at the site of the mini-implants in the case of canine retraction. This could be due to the greater extension of the elastic chains that start from the mini-implants to the high copper hook welded to the wire bracket in the en- masse retraction group. As a result, the elastic chains exerted constant pressure on the gingiva, especially at the corners of the maxilla, which led to embedding in the soft tissue surrounding them in some cases.

Chewing difficulty

The en-masse retraction caused some of the patients to experience moderate or severe chewing difficulty; the percentage of patients experiencing this difficulty was 68.42% at 24 hours of application. This discomfort was caused by the pain and swelling in the surrounding tissues of the application, as well as due to the uncomfortable contact between the cheeks with two protruding implants. This percentage decreased significantly over the following six days and increased again on the seventh day due to the continuous contact between the cheeks and the elastic chains extended from the mini-implant to the welded hooks, as well as the pain experienced by most patients after the application of retraction forces; however, this discomfort began to disappear completely or became minimal in all patients within one month of the application.

With regard to chewing difficulty with traditional anchorage, the percentage of patients experiencing no or mild difficulties was 89.47% at T1 (24 hours of mini-implant placement). This can be explained by the fact that the patients’ tongues became habituated and adapted to the chewing and swallowing processes in the presence of TPAs in their mouths since the beginning of the treatment. Then, their chewing difficulties increased on the seventh day, reaching mild to moderate levels in 78.95% of the patients due to the pain caused by retraction forces. This is consistent with the study of Kuroda et al. [22], who found a high correlation between chewing difficulty and the degree of swelling, as determined by the Rowe-Spearman correlation coefficient (P < 0.001).

The study results are also in agreement with Alfawal et al. [27], in which, through questionnaires, the patients reported that they experienced mild to moderate chewing difficulties after canine retraction using TPAs.

Speech discomfort

More than 50% of the patients reported moderate to severe discomfort in speech 24 hours after the placement of the mini-implants. The reason for this is the same reasons as those that caused the chewing difficulties, as well as the placement of the welded hooks in the anterior region and the contact of the hooks with the lip. Over the next four time points, this percentage decreased significantly, as the patients had become habituated and adapted to the presence of the mini-implants and welded hooks, and as a result, these disturbances became minimal in most patients after two weeks of use. This study result is consistent with the study by Gunduz et al. [28], who found that most patients experienced discomfort during speech due to the structures connecting the palatal implants and the supporting teeth rather than the implants themselves [28].

Cleaning difficulty

On comparing the results of the two intervention groups, it was found that the difficulty in cleaning around the mini-implants was initially greater as compared to the TPAs. This may be due to the feeling of pain and swelling in the tissues around the mini-implants and the large number of elements that contribute to the accumulation of food debris, such as braided wire, elastic chains, and hooks welded onto the archwire. However, these differences between the two groups gradually decreased and became insignificant after one month of the application. This study result differs from that of Lee et al. [20], who showed that 86% of the patients complained of the accumulation of food debris around the mini-implants after one month of the application [20]. In contrast, in this study, cleaning became quite easy for the majority of the patients within one month of starting the treatment.

Study limitations

Although this study is the first to compare pain, discomfort, and functional impairments in the case of anterior teeth retraction using two anchorage methods, some limitations were encountered. The assessment was based on patient responses to the questionnaire at different assessment time points. Several factors may have impacted their perceptions at the different activation points in the study. Further, gender-based comparisons were not conducted. Additionally, an assessment of different temporary anchorage devices placed at different regions in the oral cavity should be performed in future research.

## Conclusions

A large percentage of patients complained of pain and swelling during the first week of mini-implant placement for absolute anchorage through the en masse retraction; the pain and swelling gradually and significantly decreased within a month. The difficulties of cleaning, chewing, and speaking in the presence of mini-implants were temporary and largely disappeared within two weeks of the procedure. TPAs, when used for anchorage with the two-step retraction technique, were less problematic than mini-implants; the sensation of pain or swelling around TPAs did not last for more than a week. The TPAs caused a noticeable disturbance in chewing and speaking functions. These functional impairments gradually and significantly decreased within one week of the retraction. Approximately 40% of patients initially experienced little difficulty cleaning the areas around the TPAs, which quickly diminished within one week of the retraction.

## Figures and Tables

**Figure 4:**
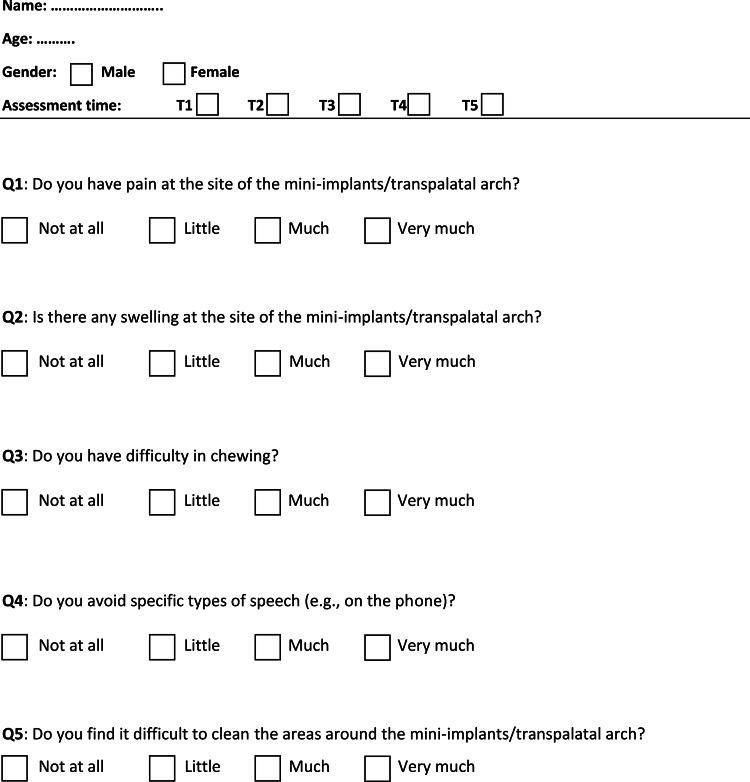
Appendix 1: The questionnaire used in the current study with its five questions.
